# Stage at diagnosis and early mortality from cancer in England

**DOI:** 10.1038/bjc.2015.49

**Published:** 2015-03-03

**Authors:** S McPhail, S Johnson, D Greenberg, M Peake, B Rous

**Affiliations:** 1 National Cancer Intelligence Network (NCIN), Public Health England, 5th Floor, Wellington House, 135-155 Waterloo Road, London SE1 8UG, UK; 2 National Cancer Registration Service (NCRS), Public Health England, Unit C, Magog Court, Shelford Bottom, Hinton Way, Cambridge CB22 3AD, UK

**Keywords:** survival, stage, mortality, early diagnosis

## Abstract

**Background:**

Stage at diagnosis is a key predictor of overall cancer outcome. For the first time, stage completeness is high enough for robust analysis for the whole of England.

**Methods:**

We analysed data from the National Cancer Registration Service's (NCRS) Cancer Analysis System on persons diagnosed with breast, colorectal, lung, prostate or ovarian cancers in England in 2012. One-year relative survival (followed-up to the end of 2013) was calculated along with adjusted excess rate ratios, for mortality within 1 year.

**Results:**

One-year relative survival decreased with increasing stage at diagnosis. For breast, prostate and colorectal cancers survival showed a major reduction for stage 4 cancers, whereas for lung and ovarian cancers there were substantial decreases in relative survival for each level of increase in stage. Excess rate ratios for mortality within 1 year of diagnosis showed that stage and age were the most important cofactors, but they also identified the statistically significant effects of sex, income deprivation and geographic area of residence.

**Conclusions:**

Further reductions in mortality may be most effectively achieved by diagnosing all cancers before they progress to stage 4, but for lung and ovarian cancers there is also a need for a stage shift to earlier stages together with efforts to improve stage-specific survival at all stages.

Improving cancer survival is a key challenge identified in ‘Improving outcomes: a strategy for cancer (Department of Health, 2011)'. Cancer survival estimates in England currently fall below those in many European countries across most cancer types (Richards, 2007; Verdecchia *et al*, 2007; Coleman *et al*, 2011; De Angelis *et al* 2014). It has been estimated that if cancer survival in England was made comparable with the European average, then 5000 or more deaths within 5 years of diagnosis could be avoided annually (Abdel-Rahman *et al*, 2009; Richards 2009a). However, if analyses are restricted to include only those who survive at least a year from diagnosis, then the difference in conditional 5-year survival between England and European countries is, in general, smaller (Thomson and Forman, 2009; Holmberg *et al*, 2010). This would suggest that differences in 1-year survival are an important driver of differences in longer-term survival.

Stage at diagnosis is highly predictive of cancer mortality, and a possible explanation for the difference in cancer survival between England and Europe is that a higher proportion of patients are diagnosed at a later stage in England (Sant *et al*, 2003; Foot and Harrison, 2011; Walters *et al*, 2013a, 2013b). The completeness of stage at diagnosis for cancers registered in England by the NCRS has improved greatly in recent years. Staging completeness now exceeds 80% for several major cancers (including breast, colorectal, lung, ovarian and prostate) diagnosed in 2012, allowing more robust analyses than have previously been possible. The remainder of staging data may be missing for various reasons: certain morphological tumour types have no formal agreed staging system; it was clinically inappropriate to stage the patient; diagnosis and/or treatment was outside the National Health Service; the patient died before staging was complete; or staging information was not transferred to the NCRS.

In England, a National Awareness and Early Diagnosis Initiative was established in 2008 (Richards, 2009b) as a joint initiative between the Government and Cancer Research UK. Much of its work has focussed on ways of promoting awareness of the early symptoms of cancer to patients and primary-care physicians. The ability to measure stage at diagnosis at a population level is vital to study the impact of such initiatives, study the scale and nature of variation in stage at diagnosis within England and to enable international comparisons.

The purpose of this study is to characterise the stage at presentation for major cancers, which have the highest recorded stage completeness, and to examine the relationship between stage at diagnosis, early mortality and major demographic variables.

## Materials and methods

Details of 156 131 malignant breast, colorectal, lung, prostate and ovarian (ICD-10 C50, C18-20, C34, C61 and C56) tumours diagnosed in 2012 in residents of England were extracted from the NCRS registration data set. Of these, 2663 cases were excluded on the basis that they were a death certificate only registration. Other exclusions comprised the following: 281 male breast cancers; 168 aged under 15 years or over 99 years at diagnosis; 187 recorded as stage 0 – for breast cancer – this is Paget's disease of the nipple, included under ICD-10 C50; 9 had a misordered date of diagnosis and date of death; and 2 had a missing deprivation quintile. Information on deaths was provided by the Office for National Statistics as part of a routine data feed to NCRS, and follow-up is complete to the end of 2013. Cancers were staged according to the TNM version 7 classification, based on clinical, imaging and pathological information (Sobin *et al*, 2009). The income deprivation quintile was derived by linking each tumour to the Index of Multiple Deprivation 2010 (Communities and Local Government, 2011) using postcode at the time of diagnosis to derive the Lower Statistical Output Area of residence at diagnosis. Equal population quintiles (of the general population) were derived from the income domain score. Geographic area of residence at the time of diagnosis was defined by the strategic clinical networks (SCNs) established in England in 2013. These SCNs have populations ranging from 2.1 to 9.0 million.

Relative survival is the ratio of the observed survival in the patient cohort and the expected survival of a cohort from the general population matched by age, sex, socioeconomic deprivation and geographic region (Government Office Region). It was calculated using the *strs* programme (Dickman *et al*, 2004) with break points set at 1, 3, 6 and 12 months and using the Ederer II method. The life tables used (Cancer Research UK Cancer Survival Group, 2006) were available with background mortality up to 2009. Age-standardised relative survival was calculated using a method of Corazziari *et al*, 2004.

Observed mortality, expected mortality and person-years of exposure time were calculated using the *strs* command in Stata 12.1 (StataCorp., 2011) for the same periods as for survival. These were summed into an overall excess mortality for the year following diagnosis. This outcome measure was chosen both for simplicity of expression and because mortality in the first year of diagnosis is of wide interest; exploration of the variation in excess mortality within the first year of diagnosis is left for future work. Excess mortality rate ratios were modelled using the *glm* command as per the ‘grouped' methodology of Dickman *et al* (2004) with sex, age band, income deprivation quintile, SCN and stage as independent variables. The baseline SCN for the calculation of rate ratios was selected from one of the two median SCNs in the distribution of 1-year relative survival. Stage 4 was used as the baseline for stage at diagnosis. Interactions between variables were explored by considering further models including an interaction between each pair of variables with a likelihood ratio test performed by comparing the model with and without interactions to determine the significance of each interaction term.

## Results

### Description of cohort

A total of 152 821 newly diagnosed malignant cancers of interest, after exclusions, were diagnosed in England in 2012. Table 1 shows the number of tumours included and the proportion broken down by age, sex, income deprivation, SCN and stage at diagnosis for breast, colorectal, lung, ovarian and prostate cancers. The median age varies between 63.0 years (breast and ovarian cancer) and 70.8 (colorectal) and 71.9 (lung) years, whereas the difference in the proportions of cases occurring in the most and least deprived varies between −11% (lung cancer) and +12% (prostate cancer). There is substantial variation in the stage breakdown with cancer type: more than two-thirds of breast cancers present at stage 1 or 2 and more than two-thirds of lung cancers present at stage 3 or 4. The other three cancers are intermediate between these two distributions. Stage completeness varies between 89% (colorectal cancer) and 82% (prostate cancer).

Table 2 shows the variation in stage at diagnosis by sex, age and income deprivation (variation by SCN is included in the Supplementary Online Material and Supplementary Table 5). More men with colorectal cancer present at stage 1 compared with women (16 *vs* 14%, *P*<0.001), whereas for lung cancer slightly fewer men present at stage 1 compared with women (12 *vs* 15%, *P*<0.001). More men present with stage 4 lung cancer compared with women (50 *vs* 48%, *P*<0.001).

For all cancer types, the proportion of missing data increases with age, particularly in those aged 80+ years. The proportion of ovarian cancers diagnosed at stage 1 drops from 54.6% in those aged 15–49 years to 19.9% in those aged 70–79 years, whereas there is no statistically significant change in lung cancer. Prostate cancer is intermediate with a change from 45.7 to 33.0%. For colorectal and breast cancer a linear change is not observed, and the highest proportion of stage 1 diagnoses occur in 60–69-year-olds.

The effect of income deprivation on stage distribution is generally <2.0% between most and least deprived for colorectal and lung cancer, and not statistically significant for ovarian cancer. For breast cancer presentation at stage 1, and at unknown stage, is more common for the least deprived (*P*<0.001), whereas presentation at stages 2, 3 and 4 is more common in the most deprived (*P*<0.001). For prostate cancer, presentation at stage 2 (*P*<0.001) and unknown stage (*P*<0.05) is more common for the least deprived, whereas presentation at stages 3 (*P*<0.05) and 4 (*P*<0.001) is more common in the most deprived.

### Variation in relative survival

Table 3 shows relative survival and age-standardised relative survival broken down by the same independent variables. Age standardisation changes overall 1-year relative survival by −0.9% (breast), +1.4% (lung), +1.0% (colorectal), −6.4% (ovarian) and +0.1% (prostate).

For the non-sex-specific cancers, survival is 4.0% higher in men (colorectal cancer) and 5.6% higher in women (lung cancer) compared with the opposite sex. Age-standardised figures are 2.2% for colorectal cancer and 6.1% for lung cancer. Relative survival varies strongly with age but, depending on cancer type, either showed only a small decline up to a certain age and then a steeper decline (breast, colorectal and prostate cancers) or declined with every increment in age category (lung and ovarian cancer). Relative survival decreases with increasing income deprivation, between the least and most deprived by 1.7% (breast), 6.5% (colorectal), 2.6% (lung), 3.0% (ovarian) and 0.8% (prostate). The age-standardised figures are 2.1%, 6.7%, 4.5%, 8.6% and 0.6%, respectively.

The variation of the relative survival between SCNs has a standard deviation of 0.5% (breast), 1.3% (colorectal), 1.6% (lung), 2.6% (ovarian) and 0.8% (prostate), assuming a normal distribution across SCNs. Relative survival is reduced with increasing stage; again some cancer types show a small reduction for lower-stage categories (breast, colorectal and prostate) followed by larger reductions in higher-stage categories, whereas other cancer types (lung and ovarian) show substantial reductions for each increase in stage at diagnosis.

### Variation in excess mortality rate ratio

Table 4 shows the excess mortality rate ratio for each independent variable. For early-stage breast and stage 1–3 prostate cancers, the mortality rate ratio is close to zero (relative to the baseline case of stage 4). Of the independent variables, stage and age have the greatest influence. Women have 14% higher excess mortality for colorectal cancer and 13% lower for lung cancer than men (*P*<0.001 for both). Rate ratios increase with increasing age and increasing deprivation, and they are statistically significant for each cancer type for older ages compared with the youngest group. Except for prostate cancer, higher income deprivation is associated with higher excess mortality. There is some statistically significant variation in the rate ratios geographically, with 5 out of 50 combinations of cancer types and SCN being statistically significant at a 95% level.

### Interactions and robustness

Of the 38 possible pairwise interaction terms across the five cancer types, 19 were significant at a 95% level in a likelihood ratio test comparing the model with and without interaction terms. Of these five were between stage and SCN and due to geographic variation in excess mortality by unknown stage. Four were associated with small subcohorts and showed no clear pattern in the excess mortality. Four were significant overall but had no individual combination of joint variables that was significant. One was owing to high excess lung cancer mortality in the N58 network being concentrated in the most-deprived quintile. One interaction between sex and stage was because of colorectal cancer excess mortality being higher in women specifically for stage 3, and one interaction between sex and age was because of worse colorectal outcomes in older women compared with men. Finally, there were four significant interactions between age and stage (data shown in Supplementary Table 6) because of higher colorectal and ovarian cancer mortality in older persons with stage 3 cancer and worse lung cancer outcomes in stage 1 and stage 2 lung cancer in persons aged 80–99 and 90–99 years. The interaction was also significant for prostate cancer, but no interaction terms were individually significant (and thus this interaction is also counted above).

The effect of using life tables from 2009 was estimated to increase the reported survival by approximately 0.2% compared with an estimate of what would have resulted from using 2012 life tables. This estimate was produced by recalculating survival with 2006 life tables and assuming a linear change in background mortality from 2006 to 2012.

## Discussion

The results presented here demonstrate the value of the substantial improvement in the completeness of staging data collected by the NCRS in England. Early-stage presentation is more likely in younger persons for ovarian and prostate cancers, and for screening age for colorectal and breast cancers. Early-stage presentation is (marginally) less likely in the more income-deprived. The analysis clarifies the expected patterns of survival, and it shows that age and stage have the greatest association on the absolute value of the 1-year survival and the adjusted excess mortality rate ratio for early mortality, whereas for sex, income deprivation and geographic area of residence the impact is smaller.

For sex, the fact that the rate ratios are close to unity implies that some of the difference in relative survival by sex is driven by age and stage case-mix, concordant with earlier work (Riaz *et al*, 2013). Excess mortality rate ratios between the least and most deprived of up to 1.4 are seen, and in colorectal cancer the associated difference in relative survival is 6.5%. This rate ratio is broadly in agreement with previously calculated mortality rate ratios of ∼1.1 per increment in income deprivation quintile (McPhail *et al*, 2013) but could also be influenced by variables outside the model, including comorbidity, differential uptake of potentially curative treatment (Peake, 2014) and the frequency of emergency presentation, all of which are higher in the more income-deprived.

Examination of the interaction between independent variables considered shows worse outcomes in stage 3 colorectal cancers in women. The relationship between age and stage in colorectal and ovarian cancers (with outcomes worse for stage 3 in older persons) and lung cancer (with outcomes worse for early stages in older persons) may indicate opportunities for re-evaluation of clinical pathways. Geographic variation in the mortality rate for unknown stage cancers is also observed, although this is likely owing to varying stage completeness.

Several SCNs show excess mortality rate ratios that are above unity and statistically significant at a 95% level. This may reflect variation not captured by the model, for example, owing to varying comorbidity, route of presentation or treatment, although, owing to the multiple testing performed, some may be simple random variations. There is significant scope for more work to describe this variation down to much smaller geographical and even health-care provider level, which is likely to be more able to help understand the reasons for such variation.

Major strengths of this study are the high stage completeness, between 80 and 90 percent, and that the data cover the whole population of England. There are four principal limitations of the study. First, the unavailability for this study of route to diagnosis, previously shown to affect short-term survival (McPhail *et al*, 2013), means that some of the excess mortality attributed to older age and higher stage might be a result of differences in route of presentation. However, McPhail *et al* (2013) also found age and stage to be the most predictive independent variables. Second, the study is limited to a single year of data, 2012, complicating the interpretation of the data in comparison with earlier studies. Additionally, during the processing of data from 2012 the registration function of the previous eight regional cancer registries merged to form the National Cancer Registration Service. Standardisation of practice can be expected in the future to improve stage completeness to a consistent level nationally, but some bias in completeness with geography still exists. Third, the model does not capture data on comorbidities. However, the influence of comorbidity on short-term mortality is lower than that of age and stage (McPhail *et al*, 2013), and thus this is unlikely to change the main conclusions of the study. Last, the outcome measured is the excess mortality in the year after diagnosis; although this is calculated by summing the excess mortality across four periods in the year, it does not attempt to characterise any non-proportionality of hazards in this period.

The Office for National Statistics publishes yearly overall cancer survival figures for a number of cancers, currently complete to tumours diagnosed up to 2011 (Solomon *et al*, 2013) and predicted for tumours diagnosed up to 2013 (Solomon *et al*, 2014). Direct comparison with these is complicated by differences in methodology, but for colon and breast (and also for oesophagus and stomach cancers – shown in Supplementary Online Material) cancers the agreement is good – ∼2% or less between years and generally 1% or less for a direct comparison of 2011. There is a notable difference in lung cancer, with overall relative survival reported here being larger (33.4 in men and 39.4 in women, 2012) than those reported by Office for National Statistics (ONS) (31.6 in men and 34.7 in women, 2011). However, ONS predictions for 2013 are larger again than figures reported here (36.1 in men, 42.2 in women, 2013, predicted). It is possible that the difference may be an artefact explicable by a change in practice in the recording of diagnosis date by the NCRS owing to better access to data from radiological systems and from the National Lung Cancer Audit (NLCA). However, there have been major improvements in the treatment rates for lung cancer, particularly in surgical resection rates, between 2005 and 2012, as demonstrated by the NLCA (Health and Social Care Information Centre, 2013). Khakwani *et al* (2013), using NLCA data, showed a significant fall in the hazard ratio of death in early-stage lung cancer between 2005 and 2010, and more recent preliminary data from the NLCA also demonstrates an improvement in overall median and one-year survival for lung cancer patients between 2010 and 2013 (MD Peake, 2014, personal communication), supporting the increase observed here.

Survival by stage has been previously published for the UK for cancers diagnosed in 2004–07 (Maringe *et al*, 2012, 2013; Walters *et al*, 2013a, 2013b). Again, direct comparison is complicated by differences in methodology and the differing definition of the tumour cohorts. However, it appears that breast and colon cancers exhibit the largest improvement in stage-specific survival for later stage cancers, whereas lung cancer has greater improvements for earlier-stage cancers. Ovarian cancer shows little change in stage-specific survival with the exception of unknown stage, which shows an improvement in all cancers compared. This increase in the survival of unknown cases is consistent with a reduction in the proportion of unknown cases that are of advanced stage.

### Implications

The results presented here support the work underpinning campaigns promoting early diagnosis, with survival estimates shown to be better for the cancers diagnosed at earlier stage. For all cancer types examined, diagnosis before stage 4 substantially increases the 1-year survival. For lung and ovarian cancer, any shift to all lower stages at diagnosis brings substantial benefit. For these two latter cancer types, there is also scope for increasing the early-stage-specific survival both by the development of more effective treatments and by ensuring the universal application of best current practice to all suitable patients, in other words reducing the large variations in the standards of care that are known to exist (Health and Social Care Information Centre, 2013).

In conclusion, the completeness of stage at diagnosis will allow more accurate comparisons between England and other countries. It will also allow the frequency of early diagnosis to be investigated more comprehensively, to examine regional and local variations and to enable better assessment of the campaigns aimed at promoting the earlier diagnosis of cancer.

## Figures and Tables

**Table 1:** Tumour cohort broken down by cancer type, sex, age, income deprivation, strategic clinical network and recorded stage

	**Breast**		**Colorectal**		**Lung**		**Ovarian**		**Prostate**	
	***n***	**%**	***n***	**%**	***n***	**%**	***n***	**%**	***n***	**%**
All	42 071	100	34 011	100	34 997	100	5455	100	36 287	100
**Sex**										
Male	0	0	19 215	56.5	19 120	54.6	0	0	36 287	100
Female	42 071	100	14 796	43.5	15 877	45.4	5455	100	0	0
**Age (years)**										
Median	63.0		70.8		71.9		63.0		70.8	
15–49	8504	20.2	2071	6.1	973	2.8	1077	19.7	405	1.1
50–59	8881	21.1	3713	10.9	3445	9.8	956	17.5	3727	10.3
60–69	11 002	26.2	8501	25.0	9583	27.4	1369	25.1	12 691	35.0
70–79	7193	17.1	10 493	30.9	11 937	34.1	1223	22.4	12 887	35.5
80–89	5307	12.6	7857	23.1	7867	22.5	720	13.2	5795	16.0
90–99	1184	2.8	1376	4.0	1192	3.4	110	2.0	782	2.2
**Income deprivation**										
Least deprived	9474	22.5	7306	21.5	4723	13.5	1089	20.0	8661	23.9
Quintile 2	9573	22.8	7488	22.0	6370	18.2	1225	22.5	8904	24.5
Quintile 3	8976	21.3	7298	21.5	7115	20.3	1177	21.6	7704	21.2
Quintile 4	7788	18.5	6488	19.1	7961	22.7	1060	19.4	6197	17.1
Most deprived	6260	14.9	5431	16.0	8828	25.2	904	16.6	4821	13.3
**Strategic clinical network**										
N50	2148	5.1	1804	5.3	2285	6.5	300	5.5	1831	5.0
N51	3233	7.7	2820	8.3	3522	10.1	479	8.8	2772	7.6
N52	2553	6.1	2233	6.6	2975	8.5	335	6.1	2036	5.6
N53	4078	9.7	3362	9.9	4299	12.3	533	9.8	3381	9.3
N54	5042	12.0	4039	11.9	3809	10.9	689	12.6	4705	13.0
N55	3874	9.2	3088	9.1	2985	8.5	522	9.6	3206	8.8
N56	4464	10.6	3726	11.0	3510	10.0	594	10.9	4175	11.5
N57	4133	9.8	3431	10.1	2876	8.2	516	9.5	3648	10.1
N58	3660	8.7	3002	8.8	2682	7.7	454	8.3	3299	9.1
N59	1702	4.0	1202	3.5	936	2.7	188	3.4	1304	3.6
N60	2499	5.9	2049	6.0	1628	4.7	294	5.4	2292	6.3
N61	4685	11.1	3255	9.6	3490	10.0	551	10.1	3638	10.0
**Recorded stage**										
Stage 1	15 752	37.4	5255	15.5	4636	13.2	1711	31.4	11 896	32.8
Stage 2	14 148	33.6	8402	24.7	2640	7.5	276	5.1	6269	17.3
Stage 3	3583	8.5	9258	27.2	7012	20.0	1567	28.7	5625	15.5
Stage 4	2366	5.6	7351	21.6	17 151	49.0	929	17.0	5836	16.1
Stage NK	6222	14.8	3745	11.0	3558	10.2	972	17.8	6661	18.4

Abbreviation: NK=not known.

**Table 2:** Variation in stage at diagnosis by sex, age and income deprivation

		**Breast**		**Colorectal**		**Lung**		**Ovarian**		**Prostate**	
**Cohort**	**Stages**	***n***	**%**	***n***	**%**	***n***	**%**	***n***	**%**	***n***	**%**
**Sex**											
Female	Stage 1	15752	37.4%	2111	14.0%	2395	15.0%	1711	31.4%	—	—
Male		—	—	3144	16.0%	2241	12.0%	—	—	11896	32.8%
Female	Stage 2	14148	33.6%	3722	25.0%	1128	7.0%	276	5.1%	—	—
Male		—	—	4680	24.0%	1512	8.0%	—	—	6269	17.3%
Female	Stage 3	3583	8.5%	3922	27.0%	3097	20.0%	1567	28.7%	—	—
Male		—	—	5336	28.0%	3915	20.0%	—	—	5625	15.5%
Female	Stage 4	2366	5.6%	3215	22.0%	7618	48.0%	929	17.0%	—	—
Male		—	—	4136	22.0%	9533	50.0%	—	—	5836	16.1%
Female	Stage NK	6222	14.8%	1826	12.0%	1639	10.0%	972	17.8%	—	—
Male		—	—	1919	10.0%	1919	10.0%	—	—	6661	18.4%
**Age (years)**											
15–49	Stage 1	2545	29.9%	282	13.6%	126	12.9%	588	54.6%	185	45.7%
50–59		3927	44.2%	526	14.2%	368	10.7%	387	40.5%	1438	38.6%
60–69		5462	49.6%	1647	19.4%	1282	13.4%	389	28.4%	4618	36.4%
70–79		2493	34.7%	1777	16.9%	1704	14.3%	243	19.9%	4247	33.0%
80–89		1132	21.3%	933	11.9%	1036	13.2%	97	13.5%	1332	23.0%
90–99		193	16.3%	90	6.5%	120	10.1%	7	6.4%	76	9.7%
15–49	Stage 2	3431	40.3%	384	18.5%	68	7.0%	52	4.8%	84	20.7%
50–59		2832	31.9%	788	21.2%	233	6.8%	60	6.3%	738	19.8%
60–69		3215	29.2%	2019	23.8%	716	7.5%	74	5.4%	2497	19.7%
70–79		2478	34.5%	2836	27.0%	962	8.1%	57	4.7%	2286	17.7%
80–89		1850	34.9%	2104	26.8%	602	7.7%	30	4.2%	625	10.8%
90–99		342	28.9%	271	19.7%	59	4.9%	3	2.7%	39	5.0%
15–49	Stage 3	997	11.7%	634	30.6%	171	17.6%	199	18.5%	37	9.1%
50–59		747	8.4%	1216	32.7%	716	20.8%	240	25.1%	587	15.7%
60–69		748	6.8%	2510	29.5%	2104	22.0%	441	32.2%	2157	17.0%
70–79		633	8.8%	2856	27.2%	2420	20.3%	440	36.0%	2148	16.7%
80–89		397	7.5%	1835	23.4%	1425	18.1%	230	31.9%	632	10.9%
90–99		61	5.2%	207	15.0%	176	14.8%	17	15.5%	64	8.2%
15–49	Stage 4	363	4.3%	478	23.1%	515	52.9%	87	8.1%	36	8.9%
50–59		385	4.3%	916	24.7%	1874	54.4%	141	14.7%	409	11.0%
60–69		488	4.4%	1790	21.1%	4750	49.6%	258	18.8%	1539	12.1%
70–79		543	7.5%	2144	20.4%	5742	48.1%	257	21.0%	2086	16.2%
80–89		499	9.4%	1723	21.9%	3710	47.2%	157	21.8%	1501	25.9%
90–99		88	7.4%	300	21.8%	560	47.0%	29	26.4%	265	33.9%
15–49	Stage NK	1168	13.7%	293	14.1%	93	9.6%	151	14.0%	63	15.6%
50–59		990	11.1%	267	7.2%	254	7.4%	128	13.4%	555	14.9%
60–69		1089	9.9%	535	6.3%	731	7.6%	207	15.1%	1880	14.8%
70–79		1046	14.5%	880	8.4%	1109	9.3%	226	18.5%	2120	16.5%
80–89		1429	26.9%	1262	16.1%	1094	13.9%	206	28.6%	1705	29.4%
90–99		500	42.2%	508	36.9%	277	23.2%	54	49.1%	338	43.2%
**Income deprivation**											
Least deprived	Stage 1	3650	38.5%	1184	16.2%	615	13.0%	347	31.9%	2898	33.5%
2		3704	38.7%	1168	15.6%	842	13.2%	345	28.2%	2874	32.3%
3		3505	39.0%	1207	16.5%	924	13.0%	361	30.7%	2571	33.4%
4		2724	35.0%	918	14.1%	1062	13.3%	343	32.4%	1963	31.7%
Most deprived		2169	34.6%	778	14.3%	1193	13.5%	315	34.8%	1590	33.0%
Least deprived	Stage 2	2965	31.3%	1827	25.0%	363	7.7%	56	5.1%	1536	17.7%
2		3223	33.7%	1879	25.1%	481	7.6%	65	5.3%	1610	18.1%
3		3061	34.1%	1783	24.4%	529	7.4%	64	5.4%	1338	17.4%
4		2702	34.7%	1628	25.1%	587	7.4%	52	4.9%	1066	17.2%
Most deprived		2197	35.1%	1285	23.7%	680	7.7%	39	4.3%	719	14.9%
Least deprived	Stage 3	745	7.9%	2053	28.1%	903	19.1%	321	29.5%	1244	14.4%
2		760	7.9%	2048	27.4%	1290	20.3%	365	29.8%	1406	15.8%
3		766	8.5%	1950	26.7%	1415	19.9%	337	28.6%	1212	15.7%
4		690	8.9%	1733	26.7%	1588	19.9%	280	26.4%	1007	16.2%
Most deprived		622	9.9%	1474	27.1%	1816	20.6%	264	29.2%	756	15.7%
Least deprived	Stage 4	465	4.9%	1522	20.8%	2368	50.1%	179	16.4%	1259	14.5%
2		442	4.6%	1564	20.9%	3080	48.4%	207	16.9%	1365	15.3%
3		457	5.1%	1553	21.3%	3523	49.5%	213	18.1%	1284	16.7%
4		529	6.8%	1459	22.5%	3887	48.8%	189	17.8%	1048	16.9%
Most deprived		473	7.6%	1253	23.1%	4293	48.6%	141	15.6%	880	18.3%
Least deprived	Stage NK	1649	17.4%	720	9.9%	474	10.0%	186	17.1%	1724	19.9%
2		1444	15.1%	829	11.1%	677	10.6%	243	19.8%	1649	18.5%
3		1187	13.2%	805	11.0%	724	10.2%	202	17.2%	1299	16.9%
4		1143	14.7%	750	11.6%	837	10.5%	196	18.5%	1113	18.0%
Most deprived		799	12.8%	641	11.8%	846	9.6%	145	16.0%	876	18.2%

Abbreviation: NK=not known.

**Table 3:** Unadjusted and age-standardised relative survival by cancer type, sex, age, income deprivation, strategic clinical network and recorded stage

	**Breast**				**Colorectal**				**Lung**				**Ovarian**				**Prostate**			
	**RS**	**95% CI**	**ASRS**	**95% CI**	**RS**	**95% CI**	**ASRS**	**95% CI**	**RS**	**95% CI**	**ASRS**	**95% CI**	**RS**	**95% CI**	**ASRS**	**95% CI**	**RS**	**95% CI**	**ASRS**	**95% CI**
All	**96.4**	96.2–96.6	**95.6**	95.2–95.9	**77.7**	77.2–78.2	**79.1**	78.6–79.5	**36.3**	35.7–36.8	**37.2**	36.7–37.8	**74.7**	73.5–75.8	**68.3**	66.9–69.6	**96.6**	96.3–96.9	**96.6**	96.3–96.8
**Sex**																				
Male	**—**	**—**	**—**	**—**	**79.4**	78.8–80.0	**80.1**	79.4–80.7	**33.7**	33.0–34.4	**34.4**	78.6–79.5	**—**	**—**	**—**	33.7–35.1	**96.6**	96.3–96.9	**96.6**	96.3–96.8
Female	**96.4**	96.2–96.6	**95.6**	95.2–95.9	**75.4**	74.7–76.1	**77.9**	77.2–78.6	**39.3**	38.5–40.1	**40.6**	78.6–79.5	**74.7**	73.5–75.8	**68.3**	39.8–41.3	**—**	**—**	**—**	**—**
**Age (years)**																				
15–49	**98.5**	98.2–98.7	**—**	**—**	**88.1**	86.6–89.4	**—**	**—**	**—**	47.2–53.5	**—**	**—**	**—**	91.2–94.3	**—**	**—**	**—**	96.3–99.3	**—**	**—**
50–59	**98.3**	98.0–98.6	**—**	**—**	**86.4**	85.3–87.5	**—**	**—**	**—**	41.3–44.7	**—**	**—**	**—**	86.3–90.4	**—**	**—**	**—**	98.7–99.5	**—**	**—**
60–69	**98.0**	97.7–98.3	**—**	**—**	**85.2**	84.4–86.0	**—**	**—**	**—**	41.2–43.2	**—**	**—**	**—**	77.4–81.7	**—**	**—**	**—**	98.7–99.2	**—**	**—**
70–79	**95.1**	94.5–95.7	**—**	**—**	**79.5**	78.6–80.3	**—**	**—**	**—**	35.8–37.5	**—**	**—**	**—**	63.6–69.1	**—**	**—**	**—**	97.7–98.5	**—**	**—**
80–89	**90.6**	89.5–91.7	**—**	**—**	**65.3**	64.1–66.4	**—**	**—**	**—**	25.1–27.2	**—**	**—**	**—**	36.3–43.8	**—**	**—**	**—**	88.6–90.8	**—**	**—**
90–99	**84.5**	81.3–87.5	**—**	**—**	**44.8**	41.7–47.9	**—**	**—**	**—**	14.9–19.7	**—**	**—**	**—**	14.9–32.3	**—**	**—**	**—**	63.1–71.8	**—**	**—**
Income deprivation																				
Least deprived	**97.3**	96.9–97.7	**96.4**	95.8–97.0	**80.4**	79.4–81.4	**81.6**	80.7–82.6	**38.0**	36.6–39.4	**40.1**	38.7–41.6	**78.3**	75.7–80.7	**73.5**	70.4–76.3	**96.9**	96.4–97.4	**96.7**	96.1–97.2
Quintile 2	**96.8**	96.3–97.2	**96.2**	95.6–96.8	**78.8**	77.8–79.8	**80.6**	79.6–81.5	**37.0**	35.8–38.3	**38.5**	37.3–39.8	**73.5**	70.9–75.9	**68.4**	65.6–71.0	**96.6**	96.1–97.1	**96.5**	95.9–97.0
Quintile 3	**96.6**	96.1–97.1	**95.9**	95.2–96.5	**77.7**	76.7–78.8	**79.2**	78.2–80.2	**35.5**	34.4–36.7	**36.8**	35.6–38.0	**73.9**	71.2–76.4	**67.8**	64.8–70.7	**96.9**	96.3–97.4	**96.9**	96.3–97.4
Quintile 4	**95.3**	94.7–95.9	**94.3**	93.5–95.0	**76.3**	75.1–77.4	**77.7**	76.6–78.8	**36.2**	35.1–37.3	**36.9**	35.8–37.9	**72.6**	69.8–75.3	**65.3**	61.9–68.5	**96.3**	95.6–97.0	**96.4**	95.6–97.0
Most deprived	**95.6**	95.0–96.2	**94.3**	93.4–95.2	**74.0**	72.7–75.2	**74.9**	73.6–76.1	**35.4**	34.4–36.4	**35.6**	34.6–36.7	**75.3**	72.2–78.1	**64.8**	61.0–68.4	**96.1**	95.2–96.9	**96.1**	95.1–96.8
**Strategic clinical network**																				
N50	**95.8**	94.6–96.8	**94.8**	93.2–96.0	**77.7**	75.5–79.7	**78.3**	76.2–80.2	**36.7**	34.7–38.8	**37.3**	35.3–39.4	**73.7**	68.1–78.4	**65.9**	60.0–71.2	**95.9**	94.5–97.1	**95.6**	94.1–96.7
N51	**96.6**	95.7–97.4	**95.7**	94.4–96.7	**77.8**	76.1–79.5	**78.2**	76.5–79.8	**36.5**	34.8–38.1	**37.0**	35.4–38.7	**74.8**	70.5–78.5	**67.7**	62.8–72.1	**95.9**	94.8–96.9	**95.9**	94.7–96.8
N52	**96.0**	95.0–96.9	**95.2**	93.7–96.3	**76.1**	74.1–78.0	**77.6**	75.7–79.4	**34.7**	33.0–36.5	**35.9**	34.1–37.7	**73.1**	67.9–77.7	**66.4**	60.3–71.8	**97.3**	96.1–98.4	**96.6**	95.0–97.7
N53	**96.6**	95.8–97.3	**95.5**	94.4–96.4	**76.6**	75.1–78.2	**78.5**	77.0–79.9	**37.9**	36.4–39.4	**38.8**	37.3–40.4	**74.7**	70.7–78.3	**69.3**	64.6–73.5	**95.7**	94.7–96.6	**95.5**	94.5–96.4
N54	**95.9**	95.2–96.5	**95.1**	94.1–95.9	**76.0**	74.6–77.4	**77.8**	76.4–79.1	**35.5**	34.0–37.1	**36.7**	35.1–38.3	**74.5**	71.0–77.7	**68.9**	64.9–72.5	**96.4**	95.6–97.1	**96.7**	95.9–97.3
N55	**96.7**	95.9–97.3	**95.4**	94.3–96.4	**76.4**	74.8–78.0	**77.7**	76.1–79.2	**35.9**	34.1–37.6	**37.1**	35.3–38.9	**73.8**	69.7–77.5	**66.0**	61.3–70.3	**95.9**	94.9–96.8	**95.9**	94.9–96.7
N56	**96.0**	95.3–96.7	**95.2**	94.2–96.1	**78.2**	76.7–79.6	**79.3**	77.9–80.7	**35.8**	34.2–37.4	**37.0**	35.3–38.7	**76.5**	72.8–79.8	**68.6**	64.3–72.5	**97.8**	97.0–98.5	**97.7**	96.8–98.4
N57	**97.3**	96.6–97.9	**96.8**	95.8–97.5	**78.8**	77.3–80.2	**80.5**	79.1–81.9	**35.9**	34.1–37.7	**37.0**	35.1–38.8	**73.4**	69.2–77.1	**68.2**	63.6–72.4	**95.8**	94.8–96.6	**96.2**	95.3–97.0
N58	**97.0**	96.2–97.6	**96.3**	95.3–97.1	**77.8**	76.2–79.4	**79.7**	78.1–81.2	**33.0**	31.2–34.8	**33.9**	32.1–35.8	**73.2**	68.7–77.1	**69.0**	64.1–73.3	**97.0**	96.1–97.8	**96.9**	95.9–97.6
N59	**97.0**	95.9–97.9	**96.3**	94.5–97.5	**80.2**	77.6–82.5	**81.5**	79.1–83.7	**38.8**	35.6–42.0	**39.3**	36.0–42.5	**81.5**	75.0–86.6	**76.2**	68.4–82.3	**98.1**	96.7–99.1	**97.6**	95.8–98.6
N60	**95.8**	94.7–96.7	**94.9**	93.5–96.0	**79.0**	77.0–80.9	**81.4**	79.6–83.1	**36.0**	33.6–38.4	**37.4**	35.0–39.8	**70.8**	65.0–75.8	**66.7**	60.5–72.2	**97.0**	95.9–98.0	**97.4**	96.3–98.2
N61	**96.4**	95.7–97.0	**95.4**	94.3–96.3	**79.3**	77.8–80.8	**80.1**	78.6–81.5	**38.9**	37.2–40.5	**39.6**	37.9–41.3	**77.4**	73.5–80.7	**68.8**	63.9–73.1	**97.3**	96.5–98.0	**96.4**	95.4–97.2
**Recorded stage**																				
Stage 1	**100.0**	99.8–100.2	**99.9**	97.4–100.0	**98.1**	97.4–98.6	**98.0**	97.3–98.5	**84.0**	82.8–85.2	**85.1**	84.0–86.2	**98.1**	97.1–98.8	**96.9**	94.5–98.3	**101.2**	100.9–101.4	**101.4**	101.0–101.7
Stage 2	**99.5**	99.2–99.7	**99.5**	99.0–99.7	**93.9**	93.2–94.5	**94.6**	93.9–95.1	**69.5**	67.6–71.4	**71.0**	69.2–72.8	**88.6**	84.0–92.1	**87.5**	81.4–91.8	**100.8**	100.4–101.2	**100.8**	100.3–101.3
Stage 3	**97.3**	96.5–97.9	**96.5**	95.3–97.4	**89.1**	88.3–89.8	**89.1**	88.3–89.8	**44.6**	43.4–45.8	**45.3**	44.1–46.4	**71.3**	68.9–73.5	**66.6**	64.1–68.9	**100.9**	100.4–101.3	**100.8**	100.3–101.3
Stage 4	**66.4**	64.4–68.4	**65.3**	63.1–67.4	**42.5**	41.3–43.6	**43.8**	42.6–44.9	**17.1**	16.5–17.7	**17.6**	17.0–18.2	**51.5**	48.1–54.7	**49.5**	46.2–52.6	**82.7**	81.6–83.8	**85.1**	84.1–86.1
Stage NK	**91.3**	90.4–92.1	**91.2**	90.2–92.1	**53.5**	51.8–55.2	**60.2**	58.4–62.0	**25.5**	24.0–26.9	**28.8**	27.1–30.5	**57.0**	53.7–60.1	**55.5**	52.1–58.8	**93.0**	92.1–93.7	**94.8**	94.1–95.4

Abbreviations: ASRS=age-standardised relative survival; CI=confidence interval; NK=not known; RS=relative survival.

**Table 4:** Modelled excess mortality rate ratio within 1 year of diagnosis by cancer type, sex, age, income deprivation, strategic clinical network and recorded stage

	**Breast**			**Colorectal**			**Lung**			**Ovarian**			**Prostate**		
	**EMRR**	**95% CI**	***P***	**EMRR**	**95% CI**	***P***	**EMRR**	**95% CI**	***P***	**EMRR**	**95% CI**	***P***	**EMRR**	**95% CI**	***P***
**Sex**															
Male				**1.00**	(Ref.)	<0.001	**1.00**	(Ref.)	<0.001						
Female				**1.14**	1.08–1.19		**0.87**	0.84–0.89							
**Age (years)**															
15–49	1.00	(Ref.)	<0.001	1.00	(Ref.)	<0.001	1.00	(Ref.)	<0.001	1.00	(Ref.)	<0.001	1.00	(Ref.)	<0.001
50–59	1.25	0.98–1.59		1.30	1.12–1.52		1.27	1.15–1.40		1.29	0.96–1.74		0.67	0.27–1.65	
60–69	1.72	1.38–2.15		1.73	1.50–1.98		1.44	1.32–1.59		2.11	1.63–2.73		0.89	0.38–2.09	
70–79	2.64	2.14–3.27		2.67	2.33–3.05		1.83	1.67–2.01		3.53	2.75–4.54		1.42	0.61–3.30	
80–89	3.82	3.10–4.70		4.78	4.19–5.45		2.61	2.38–2.86		8.98	7.01–11.5		3.59	1.55–8.33	
90–99	6.22	4.76–8.11		7.00	6.02–8.13		3.50	3.13–3.91		14.48	10.5–20.0		8.83	3.77–20.7	
**Income deprivation**															
Least deprived	1.00	(Ref.)	<0.001	1.00	(Ref.)	<0.001	1.00	(Ref.)	<0.001	1.00	(Ref.)	0.004	1.00	(Ref.)	0.234
Quintile 2	1.16	0.96–1.40		1.08	1.00–1.17		1.08	1.03–1.13		1.15	0.96–1.36		1.01	0.85–1.21	
Quintile 3	1.30	1.08–1.56		1.12	1.04–1.21		1.12	1.07–1.17		1.16	0.97–1.38		0.98	0.81–1.18	
Quintile 4	1.58	1.32–1.88		1.20	1.11–1.29		1.15	1.10–1.20		1.34	1.12–1.60		1.13	0.93–1.36	
Most deprived	1.41	1.16–1.71		1.39	1.28–1.50		1.22	1.17–1.28		1.41	1.16–1.71		1.20	0.98–1.47	
**Strategic clinical network**															
N50	1.18	0.88–1.58	<0.001	1.11	0.98–1.27	<0.001	1.03	0.96–1.10	<0.001	1.27	0.96–1.70	0.015	1.07	0.80–1.45	0.004
N51	0.91	0.68–1.23		1.00	(Ref.)		1.00	(Ref.)		1.17	0.91–1.50		0.87	0.66–1.14	
N52	1.23	0.92–1.65		1.12	0.99–1.27		1.00	0.94–1.06		1.39	1.06–1.82		0.74	0.55–1.01	
N53	1.00	(Ref.)		1.07	0.95–1.19		0.93	0.88–0.98		1.00	(Ref.)		0.94	0.74–1.20	
N54	1.59	1.25–2.03		1.13	1.01–1.25		0.96	0.90–1.01		1.13	0.90–1.43		1.00	(Ref.)	
N55	1.02	0.78–1.33		1.06	0.94–1.19		0.93	0.87–0.99		1.19	0.93–1.52		0.88	0.67–1.15	
N56	1.14	0.88–1.47		0.96	0.86–1.07		0.98	0.92–1.04		0.99	0.77–1.27		0.72	0.56–0.94	
N57	0.96	0.73–1.26		1.08	0.96–1.21		0.98	0.92–1.05		0.98	0.76–1.25		1.09	0.86–1.37	
N58	0.84	0.64–1.10		1.01	0.90–1.13		1.11	1.04–1.19		1.14	0.89–1.47		0.91	0.70–1.17	
N59	0.89	0.62–1.27		0.85	0.72–1.00		1.00	0.91–1.10		0.73	0.49–1.07		0.45	0.26–0.78	
N60	1.82	1.38–2.41		1.07	0.94–1.23		0.98	0.91–1.06		0.86	0.65–1.15		0.98	0.73–1.31	
N61	0.82	0.64–1.05		0.86	0.77–0.97		0.85	0.80–0.90		0.96	0.74–1.23		1.03	0.80–1.33	
**Recorded stage**															
Stage 1	∼0.00	*	<0.001	0.02	0.01–0.03	<0.001	0.08	0.07–0.08	<0.001	0.04	0.02–0.06	<0.001	∼0.00	*	<0.001
Stage 2	0.01	0.01–0.02		0.06	0.05–0.07		0.16	0.15–0.18		0.19	0.13–0.27		∼0.00	*	
Stage 3	0.07	0.06–0.09		0.12	0.11–0.13		0.39	0.37–0.40		0.49	0.42–0.56		∼0.00	*	
Stage 4	1.00	(Ref.)		1.00	(Ref.)		1.00	(Ref.)		1.00	(Ref.)		1.00	(Ref.)	
Stage NK	0.20	0.17–0.23		0.61	0.57–0.65		0.83	0.79–0.86		0.81	0.71–0.94		0.40	0.35–0.47	

Abbreviations: CI=confidence interval; EMRR=excess mortality rate ratio; NK=not known.

*P*-values indicate significance of likelihood ratio test on the inclusion of the variable in the model. *Non-convergence in model.
